# Demography of Reintroduced Eastern Bluebirds and Brown-Headed Nuthatches

**DOI:** 10.2193/2008-009

**Published:** 2010-12-13

**Authors:** John D Lloyd, Gary L Slater, Skip Snow

**Affiliations:** Ecostudies InstituteP.O. Box 106, South Strafford, VT 05070, USA; Ecostudies InstituteP.O. Box 703, Mount Vernon, WA 98273, USA; Everglades National Park40001 State Road 9336, Homestead, FL 33034, USA

**Keywords:** brown-headed nuthatch, Eastern bluebird, Everglades, population growth, populations, Pradel model, reintroduction, *Sialia sialis*, *Sitta pusilla*

## Abstract

Species reintroductions are used commonly as a tool for conservation, but rigorous, quantitative assessments of their outcome rarely occur. Such assessments are critical for determining success of the reintroduction and for identifying management actions needed to ensure persistence of reintroduced populations. We collected 9 years of demographic data on populations of brown-headed nuthatches (Sitta pusilla) and Eastern bluebirds (*Sialia sialis*) reintroduced via translocation into Long Pine Key, Everglades National Park, Florida, USA. Realized population growth of brown-headed nuthatches was positive in the first 3 years after cessation of translocations (λ_2002_ = 1.15, SE = 0.13; λ_2003_ = 1.28, SE = 0.12; λ_2005_ = 1.32, SE = 0.20) but became negative thereafter (λ_2006_ = 0.67, SE = 0.10; λ_2007_ = 0.77, SE = 0.13). Realized growth rate for the Eastern bluebird population did not vary among years and indicated either a stable or a slowly declining population (λ = 0.92, SE = 0.04). Reintroductions were a qualified success; they resulted in the re-establishment of populations of both species, but neither population grew to the extent expected and both remained at risk of extinction.

Despite the widespread use of reintroductions to reestablish populations of native species extirpated by habitat degradation or overexploitation (Tear et al. 1993, Wolf et al. 1996), rigorous, well-documented assessments of postrein-troduction demography remain scarce (Fischer and Linden-mayer 2000). The failure to monitor demography of reintroduced populations has hindered identification of factors associated with reintroduction success and retarded progress in improving the success rate of species reintroductions (Scott and Carpenter 1987, Minckley 1995, Sarrazin and Barbault 1996, Fischer and Lindenmayer 2000). Detailed demographic studies can provide insight into basic and applied questions of population biology, including which management actions increase the likelihood of successful reintroductions.

In this study, conducted over 9 years, we assessed demography of populations of 2 bird species, Eastern bluebird *(Sialia sialis)* and brown-headed nuthatch *(Sitta pusilla)*, that were reintroduced to Everglades National Park, Florida, USA. Both species were extirpated from Everglades National Park by the mid-1950s, part of a larger wave of local bird extinctions that was triggered by the widespread elimination and degradation of south Florida's pine (slash pine; *Pinus elliottii* var. *densa*) rockland ecosystem (Snyder et al. 1990). The reintroduction of Eastern bluebirds and brown-headed nuthatches was viewed as a test of the progress made in restoring this unique, fire-dependent ecosystem, with one measure of success being the ability to re-establish self-sustaining populations of extirpated species. Our objectives were to determine whether the reintroductions resulted in self-sustaining populations and to identify management actions needed to ensure persistence of both populations.

## STUDY AREA

We collected data on a population of each species reintroduced to Long Pine Key, Everglades National Park (25.3°N, 80.7°W). Long Pine Key is an 8,100-ha upland area, of which 4,600 ha is covered by pine rockland, a fire-dependent forest ecosystem restricted to limestone outcrop-pings in southern Florida and portions of Cuba and the Bahamas (Snyder et al. 1990). Long Pine Key is the largest remaining patch of pine rockland on the Atlantic coastal ridge. The dominant canopy species in Long Pine Key was south Florida slash pine. Other plant communities embedded within the pine forest included prairie, hardwood hammock, and cypress (*Taxodium* spp.) forest. The pine forest was even-aged as a result of extensive logging in the 1930s and 1940s, and snags were abundant due to widespread tree mortality associated with Hurricane Andrew in 1992. Beginning in the mid-1990s, Everglades National Park instituted an aggressive fire management program, with a 1–3-year fire-return interval, to reduce an overdeveloped shrub and palm understory, as well as high fuel loads that accumulated after years of fire suppression and Hurricane Andrew. In 2001, the goals of the fire management program shifted from restoration to maintenance, and the target fire-return interval was lengthened to 2–4 years.

## METHODS

We first translocated brown-headed nuthatches and Eastern bluebirds to Long Pine Key during December 1997– February 1998. A prereintroduction assessment estimated that Long Pine Key, which had been the focus of intensive efforts at ecosystem restoration, could support approximately 200 breeding pairs of both species (Slater 1997). This estimate of carrying capacity was based on mean nest densities (brown-headed nuthatches: 0.04 nests/ha; Eastern bluebirds: 0.04 nests/ha) at 2 sites in Big Cypress National Preserve (25.9°N, 80.9°W), including one from which we captured individuals for translocation, and the estimated amount of suitable habitat (4,600 ha) in Long Pine Key. The estimate of carrying capacity assumed that all pine forest in Long Pine Key was suitable for both species and that carrying capacity per unit area was the same as we observed in Big Cypress National Preserve. We obtained all brown-headed nuthatches and most (76%, *n* = 47) Eastern bluebirds used in translocations from the nearest source populations, which were located in Big Cypress National Preserve approximately 40 km from the reintroduction site. We captured the remaining Eastern bluebirds at golf courses in Naples, Florida (26.1°N, 81.8°W), approximately 140 km from the reintroduction site. Despite the proximity of the source populations, we found no evidence of natural recolonization in the 4 decades between extirpation of brown-headed nuthatches and Eastern bluebirds from Everglades National Park and the start of the reintroduction program. We captured most translocated birds on their territories and moved them as pairs (78% of brown-headed nuthatches and 76% of Eastern bluebirds), although we moved some cooperatively breeding brown-headed nuthatches as groups and some bluebird pairs with their nestlings. After capture, we transported pairs or groups to the reintroduction site, placed them in outdoor aviaries constructed in appropriate habitat, and provided them with ad libitum access to food and water. We kept Eastern bluebirds in aviaries for 1–3 weeks, except for 2 pairs that nested in an aviary, which we allowed to remain inside until their young left the nest. We released Eastern bluebird pairs with nestlings after the young had fledged and were capable of sustained flight. We kept brown-headed nuthatches in aviaries for 1–7 days. We conducted additional translocations each year during December–March (brown-headed nuthatch) and February–April (Eastern bluebird) until 2001.

We collected demographic data from the reintroduced populations in each of the breeding seasons from 1998 to 2007, excluding 2004, during which we collected no data. Thus, these data cover 4 years during which we translocated individuals to Long Pine Key and 5 years posttranslocation. We collected information on reproduction and population size by locating breeding territories through a combination of randomly located point-transect surveys, systematic playback surveys, and targeted playbacks of vocalizations in areas previously used by breeding pairs and in unoccupied habitat deemed suitable. The size of the area surveyed remained constant throughout the course of the study. We expended similar levels of survey effort in areas occupied by Eastern bluebirds and brown-headed nuthatches and in areas with no previous record of occupancy by either of the 2 study species to ensure that all individuals within the study area had a nonzero probability of detection. We conducted point-transect surveys at 100 randomly located stations, with each station visited once between December and February and again between April and June of each year. We conducted systematic playback surveys by walking transects that were spaced at approximately 300-m intervals throughout Long Pine Key. An observer stopped every 100 m along each transect, broadcast a recorded vocalization of each species, and listened for responses. We did not survey portions of transects that crossed hardwood hammocks, because neither brown-headed nuthatches nor Eastern bluebirds use this forest type. We surveyed line transects twice each year. Finally, we also broadcast recorded vocalizations in areas where territories had been located in previous years and at the ecotone between glades and pine forest, along which bluebirds frequently nested. We conducted systematic playback surveys and targeted surveys during March–June of each year. Based on the estimated effective detection radius of the point-transect surveys, and assuming the effective detection radius for systematic and targeted surveys was the same as for point-transect surveys, we calculated that our effective survey area for both species was approximately 3,940 ha, or approximately 86% of the estimated extent of pine forest in Long Pine Key (M. S. Faherty, Ecostudies Institute, unpublished data).

We indexed the size of the adult population in each year by spot-mapping territories and counting the adults associated with the territory. Brown-headed nuthatches, which breed either as pairs or in cooperative groups of up to 5 individuals, maintain group territories year-round. Eastern bluebirds, although they remain in Long Pine Key year-round, did not maintain territories outside of the breeding season. Using counts of individuals on each territory that we located may have underestimated true population size because nonterritorial individuals may have gone undetected. In addition, an unknown amount of annual variation in our counts was due to variation in our ability to detect individuals in different years. For both of these reasons, our annual index of population size is best viewed as a minimum estimate of number of birds present in each year, and as a consequence we chose to base most of our inferences on results of the demographic analyses detailed below.

Once located, we monitored each territory consistently beginning in mid-February for evidence of breeding activity. Once we noted excavation and nest-building behaviors, we checked nest sites regularly until egg-laying began. During observations on brown-headed nuthatch territories, we counted number of adults present on the territory. We indexed size of the breeding group on each territory by observing (on >2 occasions) the number of adults participating in breeding activities such as cavity excavation, nest building, incubation feeding, or feeding of the nestlings. The extent of cooperative breeding by brown-headed nuthatches seems to be density-dependent, at least in south Florida, with cooperative breeding becoming more common as populations approach carrying capacity (Cox and Slater 2008). We postulated that average size of nuthatch breeding groups might reflect density-dependent pressures, with group size increasing as the population grew and approached carrying capacity. We used linear regression to examine the relationship between our annual index of population size and our annual index of mean group size for brown-headed nuthatches. For both brown-headed nuthatches and Eastern bluebirds, we determined nest status every 3–5 days until nestlings fledged or the nest failed by using a pole-mounted video camera (Tree Top Peeper™ or Nuthatch Peeper System, Sandpiper Technologies, Inc., Manteca, CA) or by conducting behavioral observations at nests, usually for <30 minutes. To minimize disturbing the birds, we only used pole-mounted cameras on nests when a change of status was imminent (i.e., clutch completion, hatching, or fledging), and we observed activity at nests through binoculars from a distance of approximately 40 m. At this distance, we found that presence of an observer had no obvious effect on adult breeding behavior (e.g., adults did not abort incubation feeding or nestling feeding attempts).

We considered a nest successful if it fledged ≥1 nestling, and we calculated breeding productivity as number of young fledged per pair per year. We determined number of young fledged by conducting 2 visits to each territory after the date on which young left the nest. Young of both species remain with their parents for up to 1 month after departing the nest (Gowaty and Plissner 1998, Withgott and Smith 1998); however, our index of productivity reflected the minimum number fledged because some individuals that were alive may have gone undetected. As a consequence, our index of breeding productivity may underestimate actual number of young fledged per pair per year. We continued to monitor territories until mid-July to determine whether renesting occurred. We calculated an index of percentage of birds breeding each year by dividing total number of birds observed breeding by total number of birds counted in the population.

We attached colored leg bands to all translocated individuals. We also banded as many nestlings and juveniles as possible in each year of the study. However, in most years we did not capture all of the birds that fledged, because not all nests were accessible and not all juveniles were relocated after they left the nest. Thus, we also captured and banded unmarked adults that we discovered while monitoring nests or during annual population counts. We captured unbanded adults in mist nets, either by luring them to the net with recorded vocalizations or, if they were attending a nest, by setting the net outside of the nest cavity. For our analyses, we considered that any individual captured or resighted during the breeding season was alive in that year.

We examined posttranslocation demography using the reverse-time, capture–recapture models of Pradel (1996), as implemented by the Pradel survival and seniority model in Program MARK (White and Burnham 1999). In particular, we used this approach to estimate seniority probability (γ_*t*+1_), which is the probability that an individual captured at time *t*+1 was a survivor from time *t*. This parameter also yields the probability that an individual was recruited to the population during the interval, or 1 − γ_*t*+1_. Using the maximum-likelihood estimate of γ_*t*+1_, based on the best-supported model from among a set of candidate models, we derived the realized growth rate of the population (γ_*t*_) and the per-capita rate of recruitment (*f_t_*), or average number of new individuals added to the population between time *t* and time *t*+1 per individual already present in the population. We estimated derived parameters as follows:


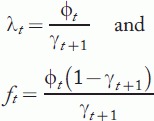


(Franklin 2001). In addition, following Nichols et al. (2000), we viewed γ as an analog of elasticity of realized γ_*t*_ to ϕ and *f*. For example, γ_*t*+1_ = 0.5 would indicate that survivors from *N_t_* and new recruits between time t and time *t*+1 made equal contributions to population growth over the interval; in contrast, γ_*t*+1_ = 0.75 would indicate that adult survival between time *t* and time *t*+1 was 3 times more important to population growth than recruitment over the interval (Nichols et al. 2000). We also examined elasticity of ϕ and *f* by calculating expected changes in λ_*t*_ as a function of proportional changes in w and *f* (Nichols et al. 2000). We estimated standard error of derived parameters using the Delta method, and we calculated approximate 95% confidence intervals (1.96 × SE) around the estimate of each derived parameter.

Our primary interest was postreintroduction demography, and we estimated γ_*t*+1_ and all derived parameters for the period 2001–2007, excluding 2004 when we collected no data. We adjusted parameter estimates to account for the unequal interval lengths by setting the length of the third interval in Program MARK to 2. We did not include in any analysis individuals that we never saw after translocation or that we saw once but that never established a breeding territory. We censored these individuals because of the bias associated with including transients in Cormack–Jolly–Seber (CJS) models (e.g., Johnston et al. 1997, Pradel et al. 1997) and because we were uncertain to what extent transience was induced by conditions at the reintroduction site versus the process of translocation itself, which was not of interest to us. Excluding transients should yield unbiased estimates of survival and capture rates for populations under study (Pradel et al. 1997). Initial attempts to estimate stage-specific (hatch-yr and ad) rates produced unreliable parameter estimates for hatch-year birds (e.g., survival and recapture rates of 0 or 1, with inestimable SEs), apparently because we had few individuals that we marked as juveniles (37 Eastern bluebirds and 19 brown-headed nuthatches). Thus, in subsequent analyses we estimated rates for the adult stage only. To do so, we used encounter histories from all individuals but censored the first survival interval for individuals banded in their hatch year. For example, we treated an individual born and marked in 2000 and resighted in 2001–2003 as if it had been marked as an adult for the first time in 2001.

We evaluated a candidate set that included 8 models: constant survival, recapture, and seniority probabilities (ϕ*p*γ); time-specific variation in one rate (ϕ_*time*_*p*γ, ϕ*p*_*time*_γ, and ϕ*p*γ_*time*_); time-specific variation in 2 rates (ϕ_*time*_*p*_*time*_γ, ϕ*p*_*time*_γ_*time*_, and ϕ_*time*_*p*γ_*time*_); and time-specific variation in all rates (ϕ_*time*_*p*_*time*_γ_*time*_). We evaluated support for each model in the reverse-time, capture-recapture analysis using Akaike's Information Criterion, as adjusted for small sample size and extrabinomial variation (QAIC_*c*_), and the quasi-likelihood-adjusted Akaike weights (*w*_*i*_) for each model in the candidate set.

General goodness-of-fit tests for the Pradel survival and seniority models cannot be implemented in Program MARK, so we estimated the extent of extrabinomial variation (*ĉ*) from the CJS model ϕ_*time*_*p*_*time*_ implemented in the live-recaptures module of Program MARK (Alisauskas et al. 2004). We used data from all years of the study, with transient individuals and hatch-year encounters censored as described for the Pradel survival and seniority analysis. We estimated *ĉ* by dividing observed *ĉ* from model ϕ_*time*_*p*_*time*_ by the mean of 1,000 simulated values of *ĉ* generated using the parametric bootstrap routine in Program MARK (White and Burnham 1999). We then used this estimate of *ĉ* to adjust model likelihoods in the Pradel survival and seniority analysis. Likelihoods differ for the CJS model and Pradel survival and seniority model because they condition on different parts of the capture history; thus, the use of a goodness-of-fit metric derived from the CJS model is not strictly appropriate as a means to account for overdispersion in the Pradel survival and seniority model. However, we believed that this was the best available approach.

We also used live-recaptures modeling of data from the entire study period to address an important assumption of the reverse-time, capture-recapture analysis, namely, that probability of recapture did not vary as a function of capture history (Franklin 2001). Permanent trap responses in capture probability can bias estimates of λ_*t*_, with trap-happy responses producing small (<0.01) to moderate (0.10) levels of positive bias in λ_*t*_ and trap-shy responses yielding a small, negative bias (Nichols and Hines 1999). To test for trap dependence in recapture probability, we first evaluated a set of models that included time-specific variation in adult survival and recapture probability (ϕ_*time*_*p*_*time*_), time-specific variation in one rate (ϕ_*time*_*p* or ϕ_*time*_), and constant adult survival and recapture probability (ϕ*p*). We evaluated support for each model using QAIC_*c*_ and *w*_*i*_. We then added a parameter to the best-supported model (or best-supported models when the top models were within 2 QAIC_*c*_ values of one another) so that initial capture probability was modeled separately from subsequent recapture probabilities. We used the relative support for this trap-dependence model to test the assumption that birds did not exhibit permanent responses to trapping. If individuals showed a positive or negative response to having been trapped, then we expected more support for the model that estimated initial capture probability separately from subsequent recapture probabilities.

## RESULTS

**Figure 1:**
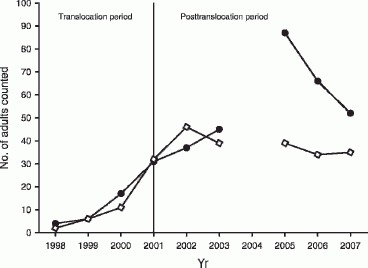
Number of documented breeding territories of brown-headed nuthatches (dark circles) and Eastern bluebirds (clear squares) reintroduced to Long Pine Key, Everglades National Park, Florida, USA, 1998–2007. We collected no data in 2004. The solid vertical line separates translocation and posttranslocation periods.

We released 47 adult brown-headed nuthatches into Long Pine Key between 1997 and 2001, 21 of which we never saw after release or we saw once but that never established a territory. Remaining birds each established a territory and were present for ≥1 year, and 16 of the 26 individuals that established a territory after translocation were present for >2 years. Annual counts of territorial adults suggested that the population grew rapidly after cessation of translocations but declined sharply from 2005 to 2007 (Fig. 1). Over the course of the study, we captured and banded 145 brown-headed nuthatches, including translocated birds. The proportion of marked individuals detected in our annual count was 100% from 1998 to 2002, but it declined to 87% in 2003 and then remained fairly constant from 2005 to 2007 (2005: 54%; 2006: 58%; and 2007: 54%). We translocated 62 Eastern bluebirds (47 ad and 15 nestlings) between 1997 and 2001. Of the 47 adults moved, we never saw 16 after release. Each of the remaining 31 established a territory. Only one of the individuals translocated as a nestling returned after fledging to establish a territory. Annual counts of territorial adults increased during the translocation period but declined gradually throughout the posttranslocation period (Fig. 1). We captured and banded 167 Eastern bluebirds during the study, including translocated individuals. The proportion of marked individuals in our annual count was 100% from 1998 to 2001, but it declined and then remained constant thereafter (2002: 85%; 2003: 85%; 2005: 62%; 2006: 88%; and 2007: 77%).

**Figure 2:**
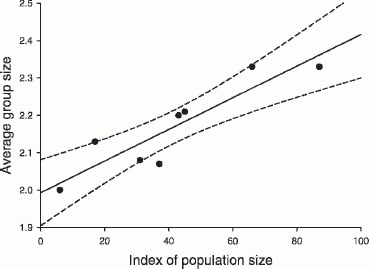
Average size of breeding groups of brown-headed nuthatches in Long Pine Key, Everglades National Park, Florida, USA, as a function of the number of adults counted in the population in each year from 1999 to 2007. Dotted lines are 95% confidence limits.

Approximately 75% of adult brown-headed nuthatches that we observed bred in a given year (95% CI = 61–90), and on average a breeding pair or breeding group fledged 1.9 young per year (95% CI = 1.6–2.3). Mean group size on each territory, averaged across years and territories, was 2.1 (range of annual means = 2.0–2.3), and average group size in a year was positively related to our index of population size in that year (r = 0.90, 95% CI = 0.55–0.98; Fig. 2). Renesting was rare among brown-headed nuthatches; only 9.1% (*n* = 13) of breeding pairs made 2 attempts in a year, and we had no evidence that brown-headed nuthatches ever made >2 nesting attempts in a season. Most renesting attempts (77%, *n* = 10) occurred after nest failure.

**Figure 3:**
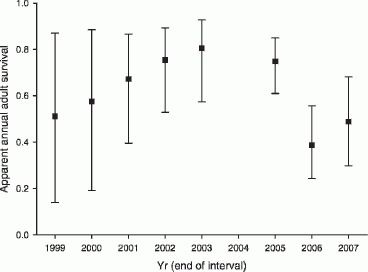
Apparent annual adult survival (±95% CI) of brown-headed nuthatches reintroduced to Long Pine Key, Everglades National Park, Florida, USA. Translocations ended in 2001, and we collected no data in 2004. We estimated apparent survival from the best-fitting model in a candidate set, ϕ_*t*_*p*.

Estimated *ĉ* from the most general model of survival (ϕ_*time*_*p*_*time*_) was 1.52. Using data from all years, we found no evidence of a permanent trap response among brown-headed nuthatches (Table 1). Support was split between the time-specific survival and constant recapture model (ϕ_*time*_*p*) and the constant survival and recapture model (ϕ*p*), and the other models received no support (Table 1). Based on the model ϕ_*time*_*p*, apparent annual adult survival was high throughout much of the study but dropped during the interval from 2005 to 2007 (Fig. 3). Recapture probability in this model was 0.96 (95% CI = 0.83–0.99). Apparent annual survival of adults under the other well-supported model, ϕ*p*, was 0.63 (95% CI = 0.55–0.71) and recapture probability was 0.94 (95% CI = 0.79–0.98).

**Table 1:** Candidate models explaining variation in apparent adult survival (ϕ) and capture probability (*p*) for brown-headed nuthatches in Everglades National Park, Florida, USA, from 1998 to 2007 (excluding 2004)

Model^a^	Model likelihood^a^	ΔQAIC_*c*_^b^,^c^	*w*_*i*_^d^	No. of parameter^e^
ϕ_*time*_*p*	191.0	0	0.44	10
ϕ*p*	206.8	0.8	0.30	3
ϕ_*time*_*p*_*trap dependence*_	191.0	2.2	0.14	11
ϕ*p*_*trap dependence*_	206.8	2.9	0.11	4
ϕ*p*_*time*_	201.0	7.8	0.01	9
ϕ_*time*_*p*_*time*_	189.0	11.8	0.00	16

a −(2 ln(*L*)/*ĉ*), where *ĉ* is a variance inflation factor calculated from the global model ϕ_*time*_ *p*_*time*_. For this model set, *ĉ* = 1.52.

b ΔQAIC_*c*_ is the difference between the value of the quasi-likelihood Akaike's Information Criterion, with a small sample correction (QAIC_*c*_), for the given model and the model with the lowest QAIC_*c*_ score.

c The lowest QAIC_*c*_ score was 212.1.

d QAIC_*c*_ wt (*w_i_*) reflects relative likelihood that the model is the best in the candidate set.

e Includes an extra parameter for *ĉ*.

In considering reverse-time models, we found strong support for constant seniority and recapture probabilities and time-dependent survival probabilities from 2001 to 2007 (Table 2). Accordingly, we used model ϕ_*time*_*p*γ to derive λ and *f* (Table 3). Estimated seniority probability (0.64; 95% CI = 0.57–0.72) was >0.5, which indicated that adult survival accounted for the most change in population size between years. Apparent adult survival was stable until the interval 2005–2007, when it dropped sharply. Estimates of λ_*t*_ (Table 3) indicated that the population grew from 2001 to 2005, with an especially large increase from 2002 to 2003, and then declined rapidly during both 2005–2006 and 2006–2007 as adult survival declined.

**Table 2:** Candidate models explaining variation in apparent adult survival (ϕ), capture probability (*p*), and seniority probability (γ) for brown-headed nuthatches in Everglades National Park, Florida, during 2001–2007 (excluding 2004)

Model^a^	Model likelihood^a^	ΔQAIC_*c*_^b^,^c^	*w*_*i*_^d^	No. of parameter^e^
ϕ_*time*_*p*γ	398.8	0	0.92	8
ϕ_*time*_*p*γ_*time*_	396.1	6.2	0.04	12
ϕ*p*γ	415.3	8.0	0.02	4
ϕ_*time*_*p*_*time*_γ	396.6	9.0	0.01	13
ϕ*p*_*time*_γ	407.8	11.2	0.01	9
ϕ_*time*_*p*_*time*_γ_*time*_	395.1	12.1	0.00	15
ϕ*p*γ_*time*_	411.4	12.6	0.00	8
ϕ*p*_*time*_γ_*time*_	405.8	18.2	0.00	13

a −(2 ln(*L*)/*ĉ*), where *ĉ* is a variance inflation factor calculated from the global model in a Cormack-Jolly-Seber analysis, ϕ_*time*_*p*_*time*_, for data collected from 1998 to 2007. For this model set, *ĉ* = 1.52.

b ΔQAIC_*c*_ is the difference between the value of the quasi-likelihood Akaike's Information Criterion, with a small sample correction (QAIC_*c*_), for the given model and the model with the lowest QAIC_*c*_ score.

c The lowest QAIC_*c*_ score was 415.5.

d QAIC_*c*_ wt (*w_i_*) reflects relative likelihood that the model is the best in the candidate set.

e Includes an extra parameter for *ĉ*.

**Table 3:** Annual estimates of apparent adult survival (ϕ) and seniority probability (γ) for brown-headed nuthatches in Everglades National Park, Florida, during 2001–2007 (excluding 2004) estimated from the reverse-time, capture–recapture model ϕ_*t*_*p*γ, and annual estimates of realized population growth (λ) and recruitment (*f*) derived from estimates of ϕ and γ

		95% CI	
		
Parameter	Estimated value	Lower	Upper
γ	0.64	0.57	0.72
ϕ_2002_	0.74	0.51	0.88
ϕ_2003_	0.82	0.60	0.94
ϕ_2005_	0.74	0.61	0.84
ϕ_2006_	0.43	0.30	0.58
ϕ_2007_	0.50	0.32	0.68
λ_2002_	1.15	0.90	1.39
λ_2003_	1.28	1.05	1.51
λ_2005_	1.32	0.92	1.71
λ_2006_	0.67	0.49	0.86
λ_2007_	0.77	0.52	1.02
*f*_2002_	0.41	0.28	0.54
*f*_2003_	0.45	0.33	0.58
*f*_2005_	0.41	0.34	0.48
*f*_2006_	0.24	0.18	0.50
*f*_2007_	0.27	0.19	0.36

We used estimated seniority probability to examine the relative effect of hypothetical changes in adult survival on population growth during the 2 years in which estimated population growth was negative. For 2005–2006, we calculated that an increase in adult survival of 77% (from the observed 0.43 to 0.76) would be required for λ_2006_ = 1.0. In 2006–2007, λ_2007_ = 1.0 would have been achieved by a 47% increase in apparent adult survival (from the observed 0.50 to 0.73). We did not use seniority probabilities to examine the response of population growth to changes in recruitment because of the deterministic relationship between adult survival and recruitment that was inherent in our best-fitting model. That is, with constant seniority probability and time-dependent survival probabilities, derived estimates of per-capita recruitment must necessarily track estimates of apparent survival. In this case, derived estimates of recruitment are a direct, linear function of apparent adult survival and therefore do not provide any additional, independent information about population dynamics.

Approximately 84% of adult Eastern bluebirds bred in a given year (95% CI = 80–88). Renesting was more common among Eastern bluebirds than brown-headed nuthatches, and 21% (*n* = 56) of all breeding pairs made 2 nest attempts in any given year. Five pairs made 3 nesting attempts in one breeding season. Most renesting attempts occurred after nest failure (*n* = 43, or 79% of all renesting attempts). Despite an increased propensity to renest, Eastern bluebirds did not fledge more young than did brown-headed nuthatches, averaging only 1.6 young per year per pair (95% CI = 1.4–1.9).

We calculated *ĉ* = 1.33 using data from all years of the study. We found evidence of permanent trap responses among Eastern bluebirds, with ϕ*p*_trap dependence_ receiving approximately twice as much support from the data as the reduced model ϕ*p* (Table 4). According to the best model, initial capture probability of Eastern bluebirds (0.95; 95% CI = 0.78–0.99) was greater than subsequent recapture probability (0.71; 95% CI = 0.40–0.90), albeit with overlapping confidence intervals, indicating moderate trap-shyness. Thus, our estimate of λ may be slightly negatively biased. Apparent adult survival from 1998 to 2007, averaged across the top 2 models, was 0.62 (unconditional 95% CI = 0.50–0.74).

**Table 4:** Candidate models explaining variation in apparent adult survival (ϕ) and capture probability (*p*) for Eastern bluebirds in Everglades National Park, Florida, USA, from 1998 to 2007 (excluding 2004)

Model^a^	Model likelihood^a^	ΔQAIC_*c*_^b^,^c^	*w*_*i*_^d^	No. of parameter^e^
ϕ*p*_*trap dependence*_	209.3	0	0.64	4
ϕ*p*	212.7	1.3	0.33	3
ϕ_*time*_*p*	204.5	8.3	0.01	10
ϕ*p*_*time*_	204.7	8.4	0.01	10
ϕ_*time*_;*p*_*time*_	194.4	12.3	0.01	16

a −(2 ln(*L*)/*ĉ*), where *ĉ* is a variance inflation factor calculated from the global model ϕ_*time*_*p*_*time*_. For this model set, *ĉ* = 1.33.

b ΔQAIC_*c*_ is the difference between the value of the quasi-likelihood Akaike's Information Criterion, with a small sample correction (QAIC_*c*_), for the given model and the model with the lowest QAIC_*c*_ score.

c The lowest QAIC_*c*_ score was 218.1.

d QAIC_*c*_ wt (*w_i_*) reflects relative likelihood that the model is the best in the candidate set.

e Includes an extra parameter for *ĉ*.

We found strong support for constant survival and seniority probabilities from 2001 to 2007, with the top 2 models differing only in whether recapture probability was constant (Table 5). Although the 2 best-supported models, ϕ*p*γ and ϕ*p*_*time*_γ, yielded nearly identical estimates for adult survival and seniority, we averaged parameter estimates across these 2 models and used unconditional estimates of standard error to account for model-selection uncertainty. The model-averaged estimate of seniority probability was 0.60 (95% CI = 0.50–0.69), suggesting that adult survival was slightly more important to population growth than recruitment. The model-averaged estimate of apparent survival was 0.55 (95% CI = 0.46–0.64), which was slightly lower than the estimate generated across all years of the study but within the estimated confidence interval. The model-averaged estimate for λ (0.92, 95% CI = 0.83–1.00) indicated that the reintroduced population of Eastern bluebirds was either stable or slowly declining from 2001–2007. Average annual per-capita recruitment to the Eastern bluebird population (*f* = 0.37, 95% CI = 0.33–0.41) was intermediate to levels estimated from the brown-headed nuthatch population. The estimated recapture probability from model ϕ*p*γ was 0.92 (95% CI = 0.76–0.98).

**Table 5:** Candidate models explaining variation in apparent adult survival (ϕ), capture probability (*p*), and seniority probability (γ) for Eastern bluebirds in Everglades National Park, Florida, during 2001–2007 (excluding 2004)

Model^a^	Model likelihood^a^	ΔQAIC_*c*_^b^,^c^	*w*_*i*_^d^	No. of parameter^e^
ϕ*p*γ	432.2	0	0.64	4
ϕ*p*_*time*_γ	423.5	2.1	0.23	9
ϕ*p*γ_*time*_	428.6	4.9	0.05	8
ϕ*p*_*time*_γ_*time*_	417.4	5.1	0.05	13
ϕ_*time*_*p*γ	430.8	7.3	0.02	8
ϕ_*time*_*p*_*time*_γ_*time*_	416.5	9.0	0.01	15
ϕ_*time*_*p*γ_*time*_	425.0	10.5	0.00	12
ϕ_*time*_*p*_*time*_γ	423.2	10.9	0.00	13

a −(2 ln(*L*)/*ĉ*), where *ĉ* is a variance inflation factor calculated from the global model in a Cormack-Jolly-Seber analysis, ϕ_*time*_*p*_*time*_, for data collected from 1998 to 2007. For this model set, *ĉ* = 1.33.

b ΔQAIC_*c*_ is the difference between the value of the quasi-likelihood Akaike's Information Criterion, with a small sample correction (QAIC_*c*_), for the given model and the model with the lowest QAIC_*c*_ score.

c The lowest QAIC_*c*_ score was 440.4.

d QAIC_*c*_ wt (*w_i_*) reflects relative likelihood that the model is the best in the candidate set.

e Includes an extra parameter for *ĉ*.

Using estimated seniority probabilities as an analogue to the elasticity of population growth rate to changes in adult survival and recruitment, we calculated that an increase in adult survival of 15% (from 0.55 to 0.70) would be required for λ = 1.0. At the same time, a 22% increase in the probability of recruitment (1−γ; from 0.40 to 0.49) would be required for λ = 1.0. Substituting this value back into the formula used to calculate per-capita recruitment, *f*, we calculated that a 22% increase in the probability of recruitment would yield *f* = 0.53, an increase of approximately 43% over the observed *f* = 0.37. Assuming constant juvenile survival, and using the average number of young fledged per year in the posttranslocation period (1.4) as a measure of baseline productivity, a 43% increase in per-capita recruitment would be achieved by increasing the average number of young fledged per year to 2.0, which is within the range of observed values.

## DISCUSSION

Although neither of the reintroduced populations grew to the extent predicted by the prereintroduction assessment, we consider the reintroduction of brown-headed nuthatches and Eastern bluebirds to Long Pine Key to be a qualified success. Armstrong and Seddon (2008) proposed that success of a reintroduction is the product of 2 discrete events: population establishment, in which population size increases from low numbers after reintroduction, and population persistence, or the ability to maintain, on average, a non-negative rate of population growth once carrying capacity has been reached. Brown-headed nuthatches and Eastern bluebirds continued to increase in number after translocations ceased, suggesting success in reestablishing populations at Long Pine Key. Less clear, however, is the ability of these populations to persist at Long Pine Key.

Insight into the likelihood of persistence may be gained by examining the possible causes of variation in postreintroduction population growth rate. In doing so, we made 2 assumptions concerning estimates of population growth rate. First, we assumed that estimates of realized λ applied to the entire population even though we included capture histories for adults only. This assumption is valid only if the age distribution is stationary or if most of the variation in population λ is due to variation in adult population size (Nichols et al. 2000). For the species that we studied, in which individuals enter the population of breeding adults within 6–9 months of birth, we suggest that the latter was the case and therefore that trends in adult λ closely approximated trends in population λ. Second, we assumed that immigration into Long Pine Key from other populations was absent or negligible and thus that changes in population size reflected processes operating within the reintroduced populations. Although we have no way to test this assumption, we believe that it is reasonable given the absence of documented records of brown-headed nuthatches or Eastern bluebirds in Long Pine Key in the 4 decades after their extirpation and preceding their reintroduction. Despite the persistence of nearby (e.g., the source populations for the reintroduction) populations, neither species was known to occur, even as vagrants, in Everglades National Park after their extirpation (see, e.g., Robertson et al. 1996). At the very least, we believe that this argues strongly that immigration played a negligible role in the dynamics of the reintroduced populations.

Given these assumptions, our interpretation of trends in postreintroduction demography depends in large part on the carrying capacity of Long Pine Key. If, as the prereintroduction assessment estimated, Long Pine Key can support 200 breeding territories of both species, then, 6 years after translocations ended, both species occurred at approximately 10% of their expected density (we counted 17 Eastern bluebird territories in 2007, and 23 brown-headed nuthatch territories). Failure of both species to show continued positive rates of population growth when existing at a small fraction of carrying capacity might indicate some systemic problem, such as inbreeding depression or Allee effects. A 6-year comparison (1998–2003) of vital rates between source populations, which we assumed had long-term rates of growth ≥1, and reintroduced populations found higher rates of survival and reproduction in the reintroduced populations, suggesting their growth was not hindered by genetic influences on vital rates (G. L. Slater, Ecostudies Institute, unpublished data). The high proportion of individuals breeding each year at Long Pine Key suggests that Allee effects were not limiting growth of reintroduced populations.

The observed patterns of population growth might also reflect Long Pine Key's position at the southeastern edge of the geographic range of both species. Peripheral populations are generally small and isolated, as is the case at Long Pine Key, and individuals in peripheral populations often are poorly adapted to the rigors of their environment and thus sensitive to even slight variations in environmental conditions (reviewed in Brown et al. 1996). As a consequence, temporal variation in population size is much greater at the edge of a species' range than in the core, and peripheral populations may be more likely to exhibit boom-and-bust cycles in response to fluctuations in abiotic conditions (Thomas et al. 1994, Curnutt et al. 1996). For example, hurricanes—2 of which, Katrina and Wilma, struck Long Pine Key in 2005—may directly cause mortality or may produce indirect effects on survival and reproduction via changes in food availability (e.g., by stripping pine trees of their cones). Other density-independent factors unique to Long Pine Key may have been important. For example, Long Pine Key is bounded on 3 sides by paved roads, and between 1999 and 2006 ≥12 hatch-year Eastern bluebirds, which often forage on the grassy roadside verges, were killed in collisions with motor vehicles. In sum, we cannot exclude the possibility that the patterns of population growth that we observed reflected the action of density-independent factors, which, in these peripheral populations, may limit the importance of density-dependent factors and reduce correlations between rates of population growth and expected carrying capacity.

Alternatively, the carrying capacity of Long Pine Key may have been overestimated during the prereintroduction assessment, in which case patterns that we observed may have reflected populations that had reached carrying capacity, albeit at a level lower than expected or desired. The prereintroduction assessment may have overestimated carrying capacity for several reasons. First, the estimate of territory density obtained from the source population was based on a small sample (*n* = 25 and *n* = 23 for brown-headed nuthatches and Eastern bluebirds, respectively) collected at 2 sites in Big Cypress National Preserve during one year (Slater 1997). However, estimates of Slater (1997) were nearly identical to those reported from a more extensive study of bird densities in slash pine forests (Land 1986, Land et al. 1989); thus, it seems unlikely that carrying capacity was overestimated because of bias in the underlying estimates of expected territory density.

Carrying capacity also may have been overestimated if, on average, habitat quality was lower at Long Pine Key, for example due to lower food abundance or increased abundance of predators or competitors. Based on point-transect surveys that we conducted between 2005 and 2008, abundance of important nest predators and competitors such as American crow (*Corvus brachyrhynchos*) and red-bellied woodpecker (*Melanerpes carolinensis*) was similar between Long Pine Key and Big Cypress National Preserve (J. D. Lloyd, Ecostudies Institute, unpublished data). We lack data on abundance of other potential predators of adults and young, such as snakes or raccoons (*Procyon lotor*), or any measures of food abundance, but trends towards higher survival and productivity at Long Pine Key suggest that, in general, conditions at the reintroduction site were roughly equivalent to those at Big Cypress National Preserve. However, variation in habitat quality may have been expressed through differences in unmeasured vital rates, such as juvenile survival.

Failure of either population to approach the expected carrying capacity of Long Pine Key also could reflect an overestimate of the amount, rather than quality, of habitat available. For example, despite efforts to impose a short fire-return interval across Long Pine Key, some areas still have dense hardwood understories, which both brown-headed nuthatches and Eastern bluebirds are known to avoid (Gowaty and Plissner 1998, Withgott and Smith 1998). Buildup of hardwood shrubs was most apparent at the ecotone between glades and pine forest, an environment which historically provided abundant nesting habitat for Eastern bluebirds. Thus, the assumption that all pine forest in Long Pine Key was suitable for both species may have been incorrect, leading to an overestimate of the number of individuals that could be supported. Under this scenario, the patterns of population growth that we observed may have reflected density-dependent limits on population growth as carrying capacity was approached. Although clearly not definitive, the correlation between the average size of breeding groups of brown-headed nuthatches and population size suggests that breeding habitat may have become saturated during years of high abundance and that second-year birds were deferring breeding in favor of remaining on their natal territories as helpers. At the same time, the notion that large parts of Long Pine Key are unsuitable for either species is somewhat perplexing given that all of the pine forest in Long Pine Key has a nearly identical disturbance history (clearcut before the establishment of Everglades National Park and then subject to the same natural [e.g., hurricanes] and anthropogenic [e.g., prescribed fire] disturbances) and thus presumably affords homogenous environmental conditions.

Our data do not allow for a reliable test of any of the preceding hypotheses. Nonetheless, action to increase the size of both populations, which were small enough to remain at risk of extinction from stochastic factors, is warranted. Given the uncertainty about factors that limit reintroduced populations, we recommend a bet-hedging strategy in which long-term management to increase suitable habitat in Long Pine Key is combined with shorter-term actions to relax potential environmental limits on key vital rates. Over the long-term, increases in the extent of breeding habitat may be achieved by continued application of prescribed fire with short return intervals (i.e., 2–3 years), especially along the ecotone between pine forest and glades and in other areas where hardwood shrubs remain dense. However, short fire-return intervals may increase the rate of snag consumption relative to the rate of snag creation, and thus Eastern bluebirds and brown-headed nuthatches, both of which nest in cavities in snags, may benefit from longer fire-return intervals (i.e., >3 years) in areas that have been burned frequently in the past. Overly frequent fire may ultimately reduce habitat availability by reducing density of large snags. Although we have no strong evidence that populations at Long Pine Key are limited by poor survival or reproduction, it may be prudent to undertake short-term measures to boost vital rates and increase population size. Apparent survival of adults had the proportionally greatest influence on population growth rate, but manipulating adult survival or emigration rates in the short term is not feasible. As such, we recommend efforts to increase breeding productivity by placing aluminum flashing above and below occupied cavities, a technique that has shown promise in excluding potential nest predators (e.g., Loeb 1996). Temporarily erecting nest boxes may also help maintain the reintroduced population of Eastern bluebirds until additional habitat becomes available. Finally, anecdotal evidence exists that juvenile survival of Eastern bluebirds might be increased via temporary reductions in speed limits on roads adjacent to Long Pine Key. During the breeding season of 2008, Everglades National Park erected temporary warning signs on one of the roads adjacent to Long Pine Key advising motorists that the area was a “bluebird crossing” and that vehicle speed should be reduced. Although we have no data regarding efficacy of the warning signs in reducing average vehicle speed, we did not document any Eastern bluebird mortality on that road in 2008, making it the first year since 1999 without ≥1 case of road mortality. As of fall 2008, Everglades National Park also reduced the posted speed limit on this road from 72 km per hour to 56 km per hour. Further study of whether warning signs and lowered speed limits reduce mortality of juvenile Eastern bluebirds is warranted.

The re-establishment of brown-headed nuthatch and Eastern bluebird populations in Long Pine Key revealed progress in restoring the pine rockland ecosystem within Everglades National Park. Occupied areas seemed to support levels of survival and reproduction sufficiently high to maintain stable populations, although we could not rule out declines in the Eastern bluebird population. However, neither population was demonstrably secure at the end of the study and continued monitoring and management of both populations is warranted. Continued restoration efforts are needed to create new breeding habitat in Long Pine Key and allow populations of both species to grow to levels that will increase likelihood of long-term persistence.

### Management Implications

Management for both species should focus on using prescribed fire to provide open forest with abundant snags (Lloyd and Slater 2007). Short-interval (1–3 years) fires are suitable in areas with dense hardwood understories, but longer return intervals should be considered in other areas so as to optimize the balance between snag creation and snag consumption. We also recommend short-term efforts to boost vital rates, especially for Eastern bluebirds, as a hedge against extinction risk.
